# Evaluating patient reported outcomes in routine practice of patients with rheumatoid arthritis treated with biological disease modifying anti rheumatic drugs (b-DMARDs)

**DOI:** 10.1186/s40064-015-1247-5

**Published:** 2015-08-28

**Authors:** Niels W. Boone, Patty Teeuwisse, Paul-Hugo van der Kuy, Rob Janknegt, Robert B. M. Landewé

**Affiliations:** Department of Clinical Pharmacy and Toxicology, Zuyderland Medical Centre, PO Box 5500, NL 6162 BG Sittard-Geleen, The Netherlands; Department of Rheumatology, Zuyderland Medical Centre, Sittard-Geleen, The Netherlands; Department of Rheumatology, Zuyderland Medical Centre, Heerlen, The Netherlands; Amsterdam Rheumatology & Immunology Centre, Amsterdam, The Netherlands

**Keywords:** Rheumatoid arthritis (RA), Routine Assessment of Patient Index Data 3 (RAPID3), Disease Activity Score 28 (DAS28), Patient reported outcome (PRO)

## Abstract

**Objectives:**

In this study the concordance between the Routine Assessment of Patient Index Data 3 (RAPID3) and the Disease Activity Score 28-joint count (DAS28) was investigated in a clinical routine outpatient setting.

**Patients and methods:**

A sample of 150 adult patients with stable RA treated with biological DMARDs (bDMARDs) was asked to complete the RAPID3 (digital or on paper) just before their outpatient routine visit during which DAS28 assessment took place. The RAPID3 correlation with and the agreement in four DAS28 categories was studied using Spearman’s rank order and Cohen’s observed kappa statistics respectively. The positive (PPV) and negative (NPV) predictive values were calculated to test whether RAPID3 could make distinction in active disease (DAS28 >3.2) or not.

**Results:**

A moderate correlation (ρ 0.576) and a poor kappa value of 0.13 were found in the whole study population. Patients reported a higher disease severity than was measured by DAS28. The PPV of RAPID3 for active disease by DAS28 was 0.59 (95 % CI 0.50–0.68) and the NPV was 0.91 (95 % CI 0.75–0.98) with a sensitivity and specificity of 96 and 40 % respectively.

**Discussion:**

While RAPID3 correlates to some extent with DAS28 at the group level, agreement between RAPID3 and DAS28 at the individual patient level is to poor to rely on RAPID3 results in monitoring patients with RA. RAPID3 tends to over-report disease activity as assessed by DAS28.

## Background

Rheumatoid arthritis (RA) is a chronic inflammatory disease that may impair daily functioning and quality of life due to pain, swelling and stiffness. The disease has an unpredictable course and the main treatment goal is to suppress disease activity in order to prevent joint damage and to improve daily living. Treatment of RA mainly includes disease modifying anti-rheumatic drugs (DMARDs) including biologicals. Both international and national treatment guidelines recommend assessing the disease activity using measures such as DAS28, and doing regular follow up assessments, in which the level of disease activity dictates the frequency of monitoring (Smolen et al. 2010; Deighton et al. 2010). Frequent collection of these patient data is challenging in a busy rheumatology practice. There are indications that the DAS28 or a quantitative joint count is not measured frequently enough in standard rheumatology care (Choy et al. 2012; Pincus and Segurado 2006).

It seems attractive to assess disease activity from the patient’s perspective using validated patient reported outcomes as an alternative or as an addition to laborious frequent joint assessments. Patient reported outcomes (PROs) are well established by groups of experts from the Outcome Measures in Rheumatoid Arthritis Clinical Trials (OMERACT)-working group (Felson et al. 1993). Patient global health (pGH) a PRO element is included as a recommendation in the 2010 ACR-EULAR guideline that proposed tighter definitions for clinical remission in clinical trials, and is now also established for clinical practice (Smolen et al. 2013). This implies that PROs have certain significance for routine clinical care.

The Routine Assessment of Patient Index Data (RAPID3) is an RA specific questionnaire on relevant patient domains including physical function, pain and global health and takes only 5 min to complete (Pincus et al. 2010). Here we have investigated by—comparing it to DAS28—whether RAPID3 is an appropriate tool to monitor disease activity and response to treatment in a real life setting.

## Methods

### Patients

This study was performed in the Atrium-Orbis medical centre hospital in Sittard, the Netherlands. The study was carried out according to the principles of the Declaration of Helsinki after ethical approval by the local ethics committee. All subjects gave their informed consent before participation in this study. Between May 2013 and April 2014 a sample was taken of consecutive adult patients with RA according to the ACR 1987 criteria that were on stable treatment with a biological DMARD (bDMARD). Clinical information such as age, sex, disease duration and medication was collected by a review of the medical records. Only patients with psychiatric illness or personality disorder were excluded.

### Measures

RAPID3 is a fully patient driven outcome measure that can be expressed as a score and is composed of the 3 PRO measures of the ACR core data set; physical function, pain and patient global estimate(Pincus et al. 2008; Castrejon and Pincus 2012; Anderson et al. 2011, 2012). Both DAS28-ESR and RAPID3 disease activity values can be distinguished into four categories. Scores are classified for DAS28-ESR of >5.1, ≥3.2 to ≤5.1, ≥2.6 to <3.2, and <2.6 and represent high, moderate, low disease activity and remission, respectively. RAPID3 scores of >12, 6.1–12, 3.1–6, and ≤3 represent high, moderate, low severity and remission, respectively ^[^(Anderson et al. 2011; Van der Heijde et al. 1993).

### Procedures

Four rheumatologists participated, and each assessed patients with rheumatoid arthritis during “real time” clinical consultations. Three qualified RA nurse practitioners (DAS28-ESR assessors) invited patients for a regular 3 monthly visit. Patients were e-mailed and asked to complete the RAPID3 questionnaire at home in the days prior the outpatient visit where the DAS28-ESR measurement was conducted. An online data portal named “Sermos E-communication in healthcare” allowed on-line availability of the patient-reported outcome questionnaire. The online data portal is protected and certified with ISO 9001/ISO 27001. Patients who were not able to complete an electronic RAPID3 completed a paper form before the outpatient visit.

### Statistical analysis

The performed RAPID3 was compared with the DAS28-ESR on a 0-30 versus 1-10 scale, respectively. DAS28-ESR and RAPID3 scores were correlated using Spearman’s rank correlation and the agreement (also for each DAS28-ESR assessor and RAPID3 method) was investigated with Cohen’s kappa coefficient per category of outcome. The following values of agreement were attributed to Cohen’s kappa: ≤0.20, poor; 0.21–0.40, fair; 0.41–0.60, average; 0.61–0.80, good, and ≥0.81, very good (Landis and Koch 1977). The positive- and negative predictive values (PPV, NPV) as well as the corresponding sensitivity and specificity of RAPID3 were calculated to test if the patient reported outcome measure could make a distinction between active and non-active disease according to DAS28-ESR criteria. SPSS Statistics software version 17 (IBM Corp., Armonk, NY, USA) was used for statistical analysis.

## Results

We have screened 1195 RA patients for the study and 293 patients met entry criteria since they were on treatment with biological DMARDs. Seventy-two patients were not willing or could not participate, and another 71 patients did not perform a RAPID3 before their DAS28-ESR assessment. Sixty-nine (46 %) of the 150 included subjects completed a RAPID3 in a conventional manner on paper and, 81 (54 %) performed it digitally via their e-mail invitation in the week before their visit. The mean age of the included patients was 60 years, 67 % were female. All patients were treated with biological DMARDs and 34 % of them were treated in combination with methotrexate. The mean levels of DAS28-ESR (3.4 ± 1.4) and RAPID3 (11.7 ± 6.2) were above the cut off levels for moderate activity and -severity according to DAS28-ESR and RAPID3 respectively. In all four DAS28-ESR disease categories the RAPID3 sub scores for pain and global health had a relatively high share in the final RAPID3 score comparing to the function scores. Demographics and patient characteristics are depicted in Table 1.

**Table 1:** Demographic and clinical patient characteristics

Characteristics	Values
Age	59.7 ± 10.8
Gender, female	100 (67)
Disease duration (year)	12.2 ± 9.1
Biological treatment duration >2 years	120 (80)
Patient measures	
Function (0–10)	2.6 ± 1.8 (total; n = 150 patients)
	4.2 ± 1.8 (DAS28-ESR >5.1; n = 22)
	3.2 ± 1.5 (DAS28-ESR ≥3.2 to ≤5.1; n = 50)
	2.4 ± 1.7 (DAS28-ESR ≥2.6 to <3.2; n = 31)
	1.4 ± 1.4 (DAS28-ESR <2.6; n = 47)
Pain (0–10)	4.4 ± 2.5 (total; n = 150 patients)
	6.6 ± 1.4 (DAS28-ESR >5.1; n = 22)
	4.8 ± 2.2 (DAS28-ESR ≥3.2 to ≤5.1; n = 50)
	4.1 ± 2.3 (DAS28-ESR ≥2.6 to <3.2; n = 31)
	3.1 ± 2.4 (DAS28-ESR <2.6; n = 47)
Patient global estimate VAS (0–10)	4.7 ± 2.5 (total; n = 150 patients)
	6.8 ± 1.2 (DAS28-ESR >5.1; n = 22)
	5.2 ± 2.3 (DAS28-ESR ≥3.2 to ≤5.1; n = 50)
	4.2 ± 2.3 (DAS28-ESR ≥2.6 to <3.2; n = 31)
	3.6 ± 2.6 (DAS28-ESR <2.6; n = 47)
Disease activity indices	
DAS28-ESR	3.4 ± 1.4
RAPID3	11.7 ± 6.2
Medications	
Etanercept (Enbrel)	61 (40.7)
Adalimumab (Humira)	53 (35.3)
Tocilizumab (Roactemra)	21 (14)
Abatacept (Orencia)	8 (5.3)
Infliximab (Remicade)	6 (4)
Certolizumab (Cimzia)	1 (0.7)
Methotrexate weekly (5–25 mg) combination therapy	34 (23)

Data expressed as mean ± SD for continuous variables and n (%) for categorical variables

*DAS28* disease activity index, *ESR* erythrocyte sedimentation rate, *RAPID3* routine assessment of patient index data 3

### Correlation between DAS28-ESR and RAPID3 scores

DAS28-ESR and RAPID3 score correlated moderately well (ρ = 0.576). The correlation coefficients between the DAS28-ESR and the individual RAPID3 components; patient physical function, pain and patient global estimate were respectively 0.569, 0.486 and 0.470, (all were statistically significant (p < 0.0001).

### DAS28-ESR and RAPID3 in categories

**Fig. 1:**
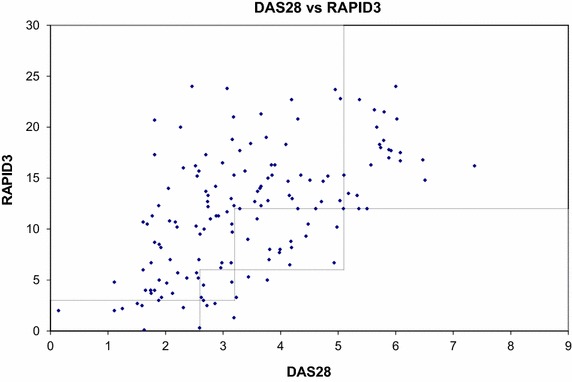
Scatterplot of DAS28-ESR and RAPID3 scores in 150 patients. The *four boxes* reflect cut off points of four categories DAS28-ESR and RAPID3; DAS28-ESR scores of >5.1, ≥3.2 to ≤5.1, ≥2.6 to <3.2, and <2.6 represent high, moderate, low disease activity and remission, respectively and RAPID3 scores of >12–30, 6.1–12, 3.1–6, and ≤3 represent high, moderate, low severity and remission, respectively. *Dots within the boxes* represent patients with a disease category match according to DAS28-ESR and RAPID3

The agreements between the RAPID3 and DAS28-ESR across categories are visualized in a scatterplot showing (mis) classifications (Fig. 1).

The calculated agreement on DAS28-ESR and RAPID3 in categories was poor; Cohen’s kappa: 0.13 (0.04–0.23), weighted kappa: 0.27. For assessor one, two and three separately agreement was similarly poor; Cohen’s kappa’s were 0.17, 0.10 and 0.11 respectively. The kappa’s for electronic and paper RAPID3 were 0.08 and 0.20 respectively and can also be stated as poor (Table 2).

**Table 2:** RAPID3 scores compared to DAS28-ESR across 4 disease categories in 150 patients

DAS28-ESR	RAPID3 scores				
	High severity	Moderate severity	Low severity	Near Remission	Total
High activity	20 (91)^a^	2 (9)	0 (0)	0 (0)	22 (15)
Moderate activity	30 (60)	17 (34)^a^	3 (6)	0 (0)	50 (33)
Low activity	13 (42)	11 (35)	3 (10)^a^	4 (13)	31 (21)
Remission	11 (23)	12 (26)	15 (32)	9 (19)^a^	47 (31)
Total	74 (49)	42 (28)	21 (14)	13 (9)	150

Cohen’s unweighted kappa’s (K) for total, individual observers and RAPID3 method (paper or electronic); K_total_ = 0.13, K_obs1 n=90_ = 0.17, K_obs2 n=31_ = 0.10

K_obs3 n=29_ = 0.11, K_electr_ = 0.08, K_paper_ = 0.20

^a^The agreement boxes for Cohen’s unweighted kappa across 4 categories. All percentages are row percentages, except total in rightmost column (column percentages)

While 96 % of the patients with a moderate to high DAS28-ESR had also a moderate to high RAPID3 score (sensitivity), only 40 % of the patients with a remission to low disease activity measured by DAS28-ESR showed remission to low disease severity according to RAPID3 (specificity).

The positive predictive value of a moderate to high RAPID3 score with regard to a moderate to high DAS28-ESR was 0.59 (95 % CI 0.50–0.68) while the negative predictive value of a low severity to remission RAPID3 with regard to finding a DAS28-ESR below 3.2 was 0.91 (95 % CI 0.75–0.98).

## Discussion

In our sample of RA patients treated with a bDMARD in common clinical practice the agreement between RAPID3 and DAS28-ESR was poor. We have found moderate associations at best, which were not clinically irrelevant. Our focus was not on finding a group-level association (which was indeed confirmed here) but rather on the level of individual agreement between two instruments developed to distinguish categories of disease activities: the DAS28 that integrates clinical and patient-reported outcome measures and the RAPID3 that is a combined score of 3 patient-reported outcomes. We have demonstrated here that there is tremendous over-call of disease activity when you rely on RAPID3 for measuring disease activity, and with DAS28-ESR as a reference. The overcall in RAPID3 disease severity could possibly be caused by comorbidities regarding the relatively high share of pain and global health scores in the final RAPID3 score across the different DAS28-ESR categories. It is obvious that this RAPID3 does not give a proper indication of DAS-measured disease activity across the four disease activity categories: Positive predictive values of RAPID3 to find active disease was moderate at best, and inappropriate to rely on in clinical practice. Negative predictive values (to find low disease activity or remission) were far better.

Introducing covariables like several DAS assessors and different methods to take RAPID3 questionnaires could have influence on the established concordance between the clinical and patient reported outcomes. However kappa values of individual assessors and RAPID3 method did not differ much and were all stated as poor. Since DAS28-ESR is not regarding tender and swollen joint counts in ankles and feet it could possibly underestimate disease severity according to RAPID3 in some patients. Moreover only relying on whatever disease parameter could be misleading in the clinical assessment of patients.

Other studies found Spearman rank correlation values in clinical routine between 0.43 and 0.91 ranging from a moderate to strong positive correlation (Pincus et al. 2010; Castrejon and Pincus 2012; Singh et al. 2012; Bossert et al. 2012; Kim et al. 2014). The Cohen`s unweighted kappa value indicative for concordance in our study is in line with kappa values found in other clinical care studies where kappa values varied between 0.16 and 0.26 and were calculated within the four disease categories (Pincus et al. 2008, 2010; Castrejon and Pincus 2012; Castrejón et al. 2013). Linear weighted kappa’s are also used in studies that addressed the agreement between RAPID3 and DAS28 and delivered relatively high kappa values 0.27 and 0.44 compared to non weighted kappa’s (Pincus et al. 2010; Castrejon and Pincus 2012; Kim et al. 2014). Only one clinical care study where 87 % of Indian patients had a DAS28-ESR >3.2 found a relatively high kappa value of 0.63 (Singh et al. 2012). Two trials found relatively high kappa values (0.25–0.36) in patient cohorts that consisted out of 73–96 % of patients with active disease (Pincus et al. 2011a, b). None of the references found observed kappa values (>0.80) providing good agreement.

Even when the RAPID3 is in poor agreement with DAS28-ESR, it is possible that RAPID3 is capable in tracking changes of DAS28-ESR score within the individual patient during longitudinal follow-up. The influence of comorbidities and disease duration on RAPID3 scoring and thereby the clinical reliability should also be topic for future research. Regarding this poor relation between the two measures in the studied population treated with bDMARDs the question is raised if the questionnaire is still up to date in patients treated with these modern agents. Differences in RAPID3 scoring in cDMARDs treated patients comparing to bDMARDs treated patients should also be addressed in future research.

## Conclusions

Our study, like others, indicates discordance in opinion between patients and physicians with respect to disease burden. This study shows a poor match in category outcomes to conclude a meaningful clinical relation between DAS28 and RAPID3 in patients treated with bDMARDs. It can therefore not substitute a frequent joint assessment. Based on our results RAPID3 is possibly useful as a non-laborious pre-screening tool to identify patients with low disease activity on a population level.
